# Competitive Revenue Strategies in the Medical Consumables Industry: Evidence from Human Resources, Research and Development Expenses and Industry Life Cycle

**DOI:** 10.3390/ijerph18063180

**Published:** 2021-03-19

**Authors:** Jianxiong Chen, Chung-Cheng Yang

**Affiliations:** Department of Accounting, National Yunlin University of Science and Technology, Yunlin 64002, Taiwan; D10825001@yuntech.edu.tw

**Keywords:** competitive advantage strategies, medical consumables industry, human resources, R&D expenses, industry life cycle

## Abstract

This study attempted to explore the competitive advantage strategies of the medical consumables industry (MCI) from the perspectives of human resources, research and development (R&D) and the industry life cycle. As one of the essential branches of modern medical device industry, the MCI has developed rapidly in recent years as global demand for medical consumables has shown continual growth, but it also faces market uncertainty. This study took Taiwan’s small/medium medical consumables enterprises (SMMCEs) as a sample, and used the translog revenue function to study the competitive advantage of the MCI through human resource and R&D investment strategies and the stage characteristics of the industry life cycle curve. The results showed that the various human resources and R&D expenses of the small/medium medical consumables industry (SMMCI) can interact with each other to influence total revenue and that the SMMCI needs more varied types of human resources to enhance its competitive advantage. The total revenue of the SMMCI decreased as education inputs rose, but it increased along with increases in the number of employee and R&D inputs. Observed from the life cycle curve of the SMMCI, total revenue increased rapidly during the startup and growth stages, increased slowly during the maturity stage, and decreased during the decline stage. Finally, we put forward competitive advantage strategies and management suggestions for medical consumables enterprises (MCEs). We are the first to document the life cycle curve and competitive advantage strategies of the MCI, thereby contributing to the related literature.

## 1. Introduction

The objective of this study was to explore the influence of human resources, R&D expenses and the industry life cycle on the revenue of the medical consumables industry (MCI). Efficient human resource management is considered to be one of the important mechanisms that enhances the competitiveness of an enterprise [1], but there is still a long-standing debate in academics and practice over the impacts of human resources. Previous studies have analyzed the influence of human resource efficiency on revenue and expenses [2]. Kimes [3] discussed key human resource issues pertaining to revenue management, and Mohsin’s [4] study linked the influence of an enterprise’s human resources services to its revenue. However, while prior studies have extensively discussed the influence of human resources on revenue, to the best of our knowledge, only Oke et al. [5] have discussed the influence of the interactions between human resources and the execution of an innovative revenue strategy. Moreover, there is no existing literature to discuss the interactive effects of human resources and R&D investment on revenue.

We expect that different human resource structures will lead to different R&D efficiencies, which in turn will affect the profitability of medical consumables enterprises (MCEs). This means that managers of MCEs need to fully consider matching R&D investment strategies when considering human resource investment strategies. After comprehensively considering the interactive effects of human resource and R&D inputs on revenue, the enterprise may have different competitive advantage strategies. In addition, the influence of the industry life cycle on enterprises is still controversial in academia and in practice. When considering the influence of the life cycle on MCEs, managers should consider not only the number of years the enterprise has been established but also its revenue. Therefore, especially for enterprises that have a declining life cycle, the development strategy of human resources and R&D inputs that are expected to increase revenue can be regarded as the key factors affecting the life cycle.

Taiwan’s medical industry and R&D technology of medical consumables are of excellent quality. According to the Global Healthcare Index published by Numbeo, Taiwan ranked among the top three in the world from 2016 to 2019. In 2020, Taiwan scored 86.71 on the Global Healthcare Index, followed by South Korea and Japan. In view of the fact that the MCI is an important driving force that promotes the development of health care, this study uses the data from Taiwan, a developed medical market, to study the competitive advantage strategies of the MCI from the perspective of human resources, R&D expenses and industry life cycle. MCEs devote a large amount of revenue to research and development each year (e.g., average annual R&D expenses by Taiwan’s small/medium medical consumables enterprises (SMMCEs) from 2009 to 2018 was NT$58.5 million), so relevant enterprises need more technical personnel to undertake the research and development of medical consumables. Such a massive recruitment of technical personnel would place strong demands on the educational training of people already working in the industry. Barney [6] pointed out that enterprises with scarce creative resources can provide strong competitive advantages for themselves: knowledge is not only a key element of human capital, but also an important element for enterprises to gain competitive advantages. Becker et al. [7] pointed out that knowledge and skills are the main manifestations of human capital. In this study, we attach great importance to the study of how human capital investment, represented by educational elements, affects the development of the MCI. In recent years, with the rapid development of the global MCI, the MCI has further expanded the number of other types of employees to meet the needs of corporate development. In addition to recruiting a large number of highly educated technical personnel for R&D, it also increased the numbers of production line personnel and managers to maintain stable operations and revenue growth. By analyzing the impact of different types of human resources and R&D expenses on revenue in the MCI, we had better understand the characteristics of the industry and promote the healthy development of the MCI. Various types of human resources in MCEs cooperate closely to maintain corporate productivity and enhance the macro- and micro-management of these enterprises. They also increase the frequency of medical consumables updates through R&D, and provide society with high-quality medical consumables. At the same time, different team structures and R&D efficiency lead to different overall enterprise efficiencies. Therefore, this study also studied whether there is an interaction between different types of human resources and R&D expenses in MCEs that affects corporate revenue.

Adizes [8] suggested that, in accordance with the industry life cycle theory, enterprises will inevitably enter the decline stage, and this industry is the same. Policy makers can extend the life cycle of an industry by making policies that are more in line with the characteristics of the industry’s life cycle. In previous research, many scholars used the industry life cycle as a control variable to analyze business strategies to improve overall efficiency [9,10,11]. By studying the characteristics of the life cycle, enterprises can carry out better operation strategies according to the stage of the industry life cycle [12,13,14]. Osman-Gani [15] believes that market competitiveness will accelerate the growth of enterprises, and may also promote the decline of enterprises. For the long-term continuous operation of enterprises, MCEs should evaluate their stage in the industry life cycle before entering the decline stage to alter their business strategies and enhance their risk management strategies [16]. The active implementation of enterprise risk management can enable managers to identify and respond to risks more efficiently [17,18,19,20]. We studied the life cycle structure and characteristics of the MCI from the perspective of revenue to evaluate its fundamentals, which will help relevant policymakers make better industrial development plans, improve the anti-risk level of the industry, and extend the industry’s life cycle.

MCEs face different market pressures and competitions in different life cycle stages and should implement different development strategies. This study focuses on a large number of SMEs in Taiwan’s MCI, which are facing more severe survival crises. We selected sample data from the Taiwan Economic Journal (TEJ) database, among which MCEs in the over-the-counter market and the emerging stock market are SMMCEs. This study used the above data and the revenue function to construct the life cycle curve of the small/medium medical consumables industry (SMMCI), and conducted an empirical analysis of MCEs to study whether the human resources and R&D expenses of SMMCEs affect their total revenue interactively. Furthermore, this article studied how R&D expenses and human resources investment, respectively, affected the revenue of the SMMCI and researched the life cycle structure and characteristics of the SMMCI. This study is the first study to draw the MCI life cycle curve through human resources, R&D expenses and revenue. We attempted to explore whether the life cycle curve of the MCI conforms to previous scholars’ research results, and whether it conforms to the general life cycle curve rules.

The healthy development of the MCI is related to national public health and plays a very important role in pandemic prevention and fair medical treatment. Therefore, it is very important to study the competitive advantage strategies of the MCI. Our theory and findings make several contributions. First, to the best of our knowledge, we pioneered the use of an MCI revenue function in industrial revenue strategy research to study the industry life cycle. There is no existing literature on the interaction of human resources and R&D expenses on revenue in this field. This article will fill this gap in the literature. Second, revenue has a significant impact on the survival and development of firms [21,22,23]. From the perspective of revenue on economic theory, by discussing the impact of human resources and R&D expenses on the revenue of the SMMCI, we find that the various human resources and R&D expenses of the SMMCI can interact with each other to influence total revenue, and the total revenue of the SMMCI decreases as education inputs increase, but it increases with increases in the number of employee and R&D inputs. Our results provide an important reference for MCEs to increase their revenue. Third, according to our research, MCI policymakers can further formulate more suitable MCI policies. We put forward specific suggestions for MCI regulators to consider. The above-mentioned ways can promote the development of SMMCEs and safeguard the interests of stakeholders.

Our findings, however, must be interpreted with caution because they are based on Taiwanese and might not be generalizable for the U.S. or other countries, due to institutional differences between Taiwan and those countries. In addition, we call for future research to investigate the impact of human resources, R&D expenses and the industry life cycle on MCEs from other perspectives, as well as cost.

The remaining chapters of this study are as follows. In Section 2, we elaborate the research background and construct the revenue function of the SMMCI through economic theory. In Section 3, we develop a number of hypotheses for this study. In Section 4, we describe the sample data and define the specific variables. In Section 5, we build the study estimation model. In Section 6, we list the empirical results. In Section 7, we summarize and describe the conclusions of this study.

## 2. Background and MCI Revenue Function

### 2.1. Background

For a long time, researchers and policymakers have been interested in related research on the industrial life cycle. A large number of studies have analyzed enterprise data in many industries. These include studies of the motor carrier [24,25,26,27], print [28], pulp and paper [29,30], semiconductor [31], mobile phone [32], telecommunication [33], lead [34], food [35,36], building [37], mining [38,39,40], forest [41], iron and steel [42,43,44], power [45,46], furniture [47], and cement [48] industries. The focus of these studies is the characteristics of the different stages of the industry life cycle, and how the life cycle of the industry can be extended using historical life cycle trends to help develop the industry.

Life cycle stages have different characteristics [12,49] as the industry faces different markets and risks; therefore, enterprises need different management and development strategies [50]. In the startup stage of corporate growth, profitability and anti-risk capabilities are weak. In the maturity stage, the enterprise has strong profitability, but this does not mean that the developmental elements are judicious. For individuals, the life cycle consumption theory links the life cycle with consumption for the first time. For enterprises, Chang and Lewins [51] believe that life cycle is closely related to revenue. Increasing revenue is one of the core goals of corporate development [52,53], The period of rising revenue usually means that the enterprise is in a more active life cycle stage, while long-term revenue decline is usually accompanied by the decline stage of the life cycle, so the promotion of revenue growth is of great significance for extending the industrial life cycle. In economics, investment in human resources can promote revenue as can investment in R&D [54], but there may be some differences between the actual situation and the theory. For different types of industries and different stages of the life cycle, some theoretical revenue factors may not be suited to a particular industry or to the current stage of its life cycle development, so these factors cannot increase industry revenue. For example, in theory increased investment in education leads to increased revenue, but because the funds of SMEs are relatively limited, investment in education may not be able to make up for the potential loss caused by the lack of investment in other fields needing urgent funding. Therefore, SMEs’ investment in education may not bring the best benefits. In addition, when an enterprise enters the decline stage of the life cycle, investment in education usually cannot have a positive effect on revenue. At present, Taiwan’s MCI has a certain degree of blind increase in investment. We can suggest that decision makers make correct factor input decisions based on the current industry conditions through the revenue function and related survey results of the industry life cycle.

As far as we know, although the previous literature has studied the life cycle of many different industries, the life cycle of the MCI has not been studied yet, let alone has its life cycle been estimated by revenue function. Research on the industry life cycle from the revenue level should enable the industry to obtain more revenue. The purpose of this study is to examine the relationship between input factors and revenue based on the research of the revenue factors and life cycle of Taiwan’s SMMCI and to estimate the characteristics of the industry’s life cycle, so as to facilitate policy makers to implement policies that are more conducive to extending industrial life by analyzing the observed situation.

### 2.2. Revenue Function of MCI

Yang, Tsai, and Fu [55] pointed out that enterprises need to maintain good business operations through reasonable allocations of human resources, which can be classified by quality and quantity. In the case of quality, corporate employees develop human capital through education, training and experience at work, thereby improving their professional level. In this study, limited by the availability of data, we used education as a proxy for human capital. In the case of quantity, MCEs in Taiwan generally have production line employees and technical personnel (classified as direct personnel) and management personnel to carry out various business and management activities. Therefore, this study used Equation (1) to describe the production function of MCEs.

(1) $$y=f\left(h,x_{1},x_{2},d_{-1}\right)$$

In this equation, *y* represents the sales of various medical consumables carried out by the MCEs; *h* is the human capital accumulated by the enterprise’s employees through education; $x_{1},x_{2}$ represent direct and managerial personnel input, and $d_{-1}$ represents the enterprise’s research and development spending over the previous year (R&D investment has a delayed effect on company revenue; that is, it usually does not have an effect in the current period. In order to identify the partial effect of R&D investment more carefully, this study used the R&D from the previous year). The outputs and inputs must be nonnegative real numbers: y≥0, h≥0, $x_{i}\geq 0$, $d_{-1}\geq 0$, and *i* = 1, 2; furthermore, the marginal product of the inputs in the production function has monotonically increasing and decreasing characteristics: ∂f(·)/∂h≥0, $\partial f\left(\cdot \right)/\partial x_{i}\geq 0$, $\partial f\left(\cdot \right)/\partial d_{-1}\geq 0$, $\partial^{2}f\left(\cdot \right)/\partial h^{2}\leq 0$, $\partial^{2}f\left(\cdot \right)/\partial x_{i}^{2}\leq 0$ and $\partial^{2}f\left(\cdot \right)/\partial d_{-1}^{2}\leq 0$.

MCEs maximize their revenue at their existing technology level, and the corporate revenue function is as follows. (One of the key points of this study was to estimate the life cycle curve of the MCI. The industry life cycle presents the changes during different years or periods starting with their establishment, and the revenue of these enterprises, not the changes of profit. In addition, research and development of an enterprise is usually represented by R&D inputs as a proxy variable. The second focus of this study was to explore the influence of R&D inputs on the future development of the enterprise, which mainly depends on whether revenue can continue to grow, so this study focused on the revenue of the enterprise):

(2) $$r\left(p;h,x_{1},x_{2},d_{-1}\right)=max\;py\;subject\;to\;y=f\left(h,x_{1},x_{2},\;d_{-1}\right)$$

In the above equation, *r* represents the total revenue of MCEs, and *p* represents the prices of consumables provided by MCEs.

General research used the Cobb–Douglas function, and the corporate revenue function as follows:

(3) $$lnr=\alpha_{0}+\delta \;ln\;p+\alpha_{1}lnh+\overset{2}{\underset{i=1}{\sum }}\beta_{i}lnx_{i}+\gamma_{1}lnd_{-1}$$

Since the model has a single output and the revenue function is characterized by the homogeneous degree 1 in output prices (*δ* = 1), this study normalized it by setting *p* = 1 [56]. Therefore, we can simplify Equation (3) as follows:

(4) $$lnr=\beta_{0}+\alpha_{1}lnh+\overset{2}{\underset{i=1}{\sum }}\beta_{i}lnx_{i}+\gamma_{1}lnd_{-1}$$

The above equation is a log–linear model. Greene [57] believes that current research on revenue profit and other functions usually uses a flexible functional form because a flexible functional form is good at analyzing some complex characteristics of the function, such as the effects of the second derivatives of function and elasticities of substitution. The most popular flexible functional form is the translog model, which is often interpreted as a second-order approximation to an unknown functional form. We set the translog revenue function of the MCI as follows [58]:

(5) $$lnr=\beta_{0}+\alpha_{1}lnh+\overset{2}{\underset{i=1}{\sum }}\beta_{i}lnx_{i}+\gamma_{1}lnd_{-1}+\frac{1}{2}\alpha_{11}{\left(lnh\right)}^{2}+\frac{1}{2}\sum_{i=1}^{2}\sum_{j=1}^{2}\beta_{ij}lnx_{i}lnx_{j}\;+\frac{1}{2}\gamma_{11}{\left(lnd_{-1}\right)}^{2}+\sum_{i=1}^{2}\theta_{1i}lnhlnx_{i}+\varepsilon_{11}lnhlnd_{-1}+\sum_{i=1}^{2}\eta_{i1}lnx_{i}lnd_{-1}$$

In the above equation, if $\alpha_{11}=\beta_{ij}=\gamma_{11}=\theta_{1i}=\varepsilon_{11}=\eta_{i1}=0$, the translog model can be reduced to a Cobb–Douglas model. 

## 3. Hypothesis Development

Efficient human resources can improve the quality of business operations [59,60,61,62] and create more profits for enterprises [63,64,65,66]. The educational background of employees is an important part of human resources [67,68,69]. Employees with different educational backgrounds have different work efficiencies and make different contributions to enterprise revenue. Employees with different educational backgrounds and different professional backgrounds will work together to form different human resource structures, and different human resource structures will show different work coordination effects, which will affect the overall performance and operation of the enterprise. MCEs can be regarded as a team, and different team member structures have an important impact on the total revenue of MCEs.

Halici et al. [70] pointed out that employee heterogeneity is an important advantage to help enterprises survive in fierce market competition. Maintaining diversity in the team means that employees with different characteristics are incorporated into the team, including employees of a different age, gender, work experience and educational background. There are diverse skills and educational backgrounds in the team. The diversity of skills and educational backgrounds in the team is the key means by which enterprises increase innovation. The heterogeneity of employees in an enterprise is often positively correlated with creative solutions because heterogeneity can have a positive impact on enterprise performance [71,72]. The diversification of corporate talents will enable enterprises to have better flexibility and perform better at marketing and strategic decision-making, thereby improving the overall performance. Human capital is an important resource for innovation [73,74,75]. There is no doubt that the heterogeneity of team members can become an important input for the enhancement of corporate human capital, which makes the enterprise more innovative and creative in the market and has a positive effect on total revenue [76]. MCE employees comprise people of different professional and educational backgrounds, they must carry out sufficient collaboration at work, which will affect the total revenue of the enterprise. Moreover, their different educational backgrounds obviously affect R&D differently. These differences affect R&D efficiency, and the employees’ contribution to total revenue is also different, so we put forward the first hypothesis of this study:

**Hypothesis** **1** (**H1**)**.** *Ceteris paribus, the various human resources and R&D expenses of the SMMCI can interact with each other to influence total revenue.*

With the rapid development of Taiwan’s medical technology in recent years, medical tourism by patients from Asia has become increasingly popular, which also makes Taiwan’s MCI have higher employee input. Crook et al. [77] conducted a multivariate analysis on the relationship between human resources and corporate performance, and proposed a positive correlation between human resources and corporate performance. Hitt et al. [67] explored the impact of human resources and performance of American law firms, and found that they were positively correlated; that is, the more employees, the better the performance. We expect that in the SMMCI, total revenue will also increase with an increase in the number of employees.

The MCI needs more technical input to update products to meet the increasing medical needs of society, and investment in technology requires R&D expenses and employees with varied educational backgrounds. Employees with high education receive adequate professional education during school, and their deeper professional knowledge can promote the development of the enterprise and help increase total revenue. The investment in R&D will further enhance the innovation ability of the enterprise, so that the company can occupy a larger market share and increase total revenue. Therefore, the following three hypotheses were formed:

**Hypothesis** **2a** **(H2a).** *Ceteris paribus, the total revenue of the SMMCI increases with increases in education inputs.*

**Hypothesis** **2b** **(H2b).** *Ceteris paribus, the total revenue of the SMMCI increases with increases in the number of employee inputs.*

**Hypothesis** **2c** **(H2c).** *Ceteris paribus, the total revenue of the SMMCI increases with increases in R&D inputs.*

There have been many studies on the enterprise life cycle theory, but no scholar has focused on the life cycle of the MCI. Haire [78] put forward the theory of enterprise life cycle based on the similarity between enterprise and biology. At different stages of the life cycle, enterprises face different conditions and management methods are not the same [79,80]. Yang and Shyu [81] took Taiwan’s electrical machinery industry as an example, and pointed out that the development strategy of an enterprise should change according to the environment in different life cycle stages. The life cycle of the MCI is also similar to the biological life cycle from birth to death: it goes through the startup stage, growth stage, maturity stage, and decline stage. The developmental characteristics of each life cycle stage are different, and the corresponding operation and management focus are also different. Abednazari and Noravesh [82] studied the corresponding investment opportunities and revenue status of enterprises in different stages, and analyzed the relationship between them. They found that the correlation between investment opportunity and revenue status of enterprises in different life cycle stages was different. In particular, compared with enterprises in the decline stage, enterprises in the growth stage had a closer correlation between investment opportunity and revenue status. We believe that for the SMMCI, there is a similar situation described above. The SMMCI is limited by the scale of having less investment in the early stage. However, due to the rise of the global medical consumables market in recent years, the MCI has developed rapidly. Under this market situation and investment, the SMMCEs have a high cost–benefit ratio, so total revenue shows a rapid growth trend in the startup and growth stages, but after the maturity stage, due to saturation and fierce competition in the medical consumables market, the growth of total revenue declines. In the decline stage, due to the aging of medical consumables technology and the lack of innovation, total revenue will enter a retrogressive stage. Therefore, the hypothesis is as follows:

**Hypothesis** **3** **(H3).** *Ceteris paribus, in the life cycle of the SMMCI, the total revenue increases rapidly during the startup and growth stages, increases slowly during the maturity stage, and decreases during the decline stage.*

## 4. Data and Variables

### 4.1. Data Source and Sample Period

The data for this study comes from the TEJ database in Taiwan. Taking into account the relevant variables and data integrity and other factors, this study selected a total of 10 years from 2009 to 2018 as the sample period, and deleted the samples with zero relevant data due to data errors or omissions; thus, the basic data of this study was closer to the actual situation of MCEs. After the deletions, the remaining data spanned 10 fiscal years and totalled 198 final observations.

In addition, given that the number of SMMCEs in Taiwan is far more than that of large-scale MCEs, they have more comprehensive influence than large-scale MCEs, and SMMCEs will face a more severe survival crises, and in order to meet the quantitative research requirements of sample data, this study only selects Taiwan’s SMMCEs as the research sample. According to Taiwan’s division of enterprise scale, we selected the over-the-counter market and emerging stock market MCEs in the TEJ database to classify as SMMCEs. The sample selection is summarized in Table 1.

### 4.2. Variable Definitions

With regard to the dependent variables, we use (*REVENUE*) to represent the total revenue (*r*) of MCEs.

We divided the independent variables in this study into three parts. The first part measured qualitative human resources; the second part measured quantitative human resources; and the third part measured R&D expenses. Firstly, we measured the accumulation of human capital by employees’ educational background. The educational level is based on the proportion of the number of employees with different educational backgrounds in the MCEs to the total number of employees ($w_{1},w_{2},{\;w}_{3},{\;w}_{4},{\;w}_{5}$), which is then multiplied by the weights of the different educational background proportions by the number of years of study in Taiwan: 12 years for high school, 14 years for junior college, 16 years for university, 18 years for master’s degree, and 22 years for doctoral degree. The result gave us the average years of education (*EDUC*) of enterprise employees. Secondly, for the measurement of corporate human resources we used the total number of direct personnel (*DIRECT*) and the total number of management personnel (*MANAGER*) as proxy variables, and the sum of the two types of employees equals the total number of employees (*EMPLOYEE*). In the third part, we considered that for the MCI, huge R&D expenses had a significant impact on the development of enterprises, so we selected the total R&D expenses (*RD*) of MCEs as one of the important independent variables.

Finally, we subtracted the opening year of the medical consumables enterprise (MCE) from the year in which the above data was located and added 1 to get the age of the MCE (*AGE*). In this study, we took the age of establishment (*AGE*) as the control variable for the study. The definitions of the above variables are summarized in Table 2.

### 4.3. Descriptive Statistics

Table 3 shows the descriptive statistics of the sample data of MCEs. We mainly reflected the size of the enterprise through total revenue (*REVENUE*) and the total number of employees (*EMPLOYEE*). From 2009 to 2018, the average of total revenue (*REVENUE*) and the total number of employees (*EMPLOYEE*) of Taiwan’s SMMCEs was greater than the median, and the gap between the two was very large, which indicated that the variable data of scale was seriously skewed to the right. Looking at the overall trend from 2009 to 2018, the gap between the average and median of the total revenue (*REVENUE*) steadily increased year by year. At the same time, the gap between the average and median of the total number of employees (*EMPLOYEE*), except for a few years, almost steadily increased year by year. The gap between the two grew larger and larger, indicating that the variable data had further expanded to the right. The main reason for this phenomenon is that among the SMMCEs in Taiwan, medium-sized MCEs occupy more and more market share, while small MCEs occupy an increasingly smaller market share.

We observed the trend of changes in scale variables from 2009 to 2018, and found that average total revenue (*REVENUE*) and the average total number of employees (*EMPLOYEE*) of SMMCEs showed a significant growth trend. Average total revenue increased by 98.83%, and the average total number of employees increased by 113.55%. However, from 2009 to 2018, the median of the total revenue of SMMCEs only increased by 6.85%, and the median of the total number of employees only increased by about 53.37%. This showed that among the medium-sized and small-sized MCEs in Taiwan, the medium-sized MCEs accounted for a larger proportion of total revenue, and the gap between the small MCEs widened. From the enterprise employee data, we found that the scale difference between medium-sized MCEs and small-sized MCEs was further expanding.

Education, like other variables, belongs to a non-normal distribution, which shows a wide peak and thin tail posture. On the whole, the average employee educational level of Taiwan’s SMMCEs from 2009 to 2018 showed an increasing trend, which also reflected the impact of the rapid expansion of Taiwan’s higher education in recent years on the MCI.

During the sample period, the R&D investment of SMMCEs in Taiwan continued to increase. From 2009 to 2018, average R&D expenses increased by NT$33.56 million, an increase of 89.86%, but the median only increased by 27.55%. It further confirmed the growing gap between medium-sized MCEs and small MCEs in Taiwan.

### 4.4. Correlation Matrix

Table 4 summarizes the Pearson correlation coefficients between variables. It can be seen from Table 4 that the higher the educational level of employees recruited by SMMCEs, the smaller the number of management personnel and indirect employees. We believe this is because the economic strength of SMMCEs is far inferior to that of large MCEs, and their limited funds need to be balanced between educational resources and the number of employees. When the investment in employees’ education is more, it will certainly reduce the investment in the number of employees, and vice versa.

From Table 4, we can see that the older an enterprise is, the more it spends on R&D. This may be due to the fact that older enterprises have greater R&D needs, which will further encourage enterprises to become bigger and stronger. In addition, for SMMCEs, the correlation coefficient between the number of years of establishment and the average years of education of employees is significantly negative because those SMMCEs have been established for a long time. The lack of talent with high-end educational backgrounds results from the fact that most of the highly educated employees that entered the MCI were recruited by large MCEs, and it was generally difficult for SMMCEs to recruit highly educated employees at that time. However, with the rapid development of higher education in Taiwan, the average educational level of employees in SMMCEs established in recent years has significantly improved.

## 5. Estimation Model

To estimate the life cycle curve of the MCI, we used the years of establishment of MCEs (*AGE*) as a control variable, which has a quadratic polynomial relation with revenue. This variable is included in the translog revenue function of the MCI in Equation (5). The translog revenue function estimator is as follows:

(6) $$\;lnREVENUE=\beta_{0}+\alpha_{1}lnHUCAP+\beta_{1}lnDIRECT+\beta_{2}lnMANAGER+\gamma_{1}lnRD_{-1}\;+\alpha_{11}{\left(lnHUCAP\right)}^{2}\;+\beta_{11}{\left(lnDIRECT\right)}^{2}+\beta_{22}{\left(lnMANAGER\right)}^{2}+\gamma_{11}{\left(lnRD_{-1}\right)}^{2}\;+\beta_{12}\left(lnDIRECT\right)(lnMANAGER)+\varepsilon_{11}(lnHUCAP)(lnRD_{-1})\;+\theta_{11}(lnHUCAP)(lnDIRECT)+\theta_{12}(lnHUCAP)(lnMANAGER)\;+\eta_{11}(lnDIRECT)(lnRD_{-1})+\eta_{21}(lnMANAGER)(lnRD_{-1})\;+\delta_{1}AGE+\delta_{2}AGE^{2}$$

To test for the existence of any interaction effect or effects from the second derivatives of the function between the independent variables, we established the following null hypothesis:

(7) $$\alpha_{11}=\beta_{ij}=\gamma_{11}=\theta_{1i}=\varepsilon_{11}=\eta_{i1}=0;\;i,\;j=1,\;2$$

If the above null hypothesis cannot be rejected, Equation (6) is reduced to the Cobb–Douglas revenue function estimator as follows:

(8) $$ln\;REVENUE=\beta_{0}+\alpha_{1}lnHUCAP+\beta_{1}lnDIRECT+\beta_{2}lnMANAGER\;+\gamma_{1}lnRD_{-1}+\delta_{1}AGE+\delta_{2}AGE^{2}$$

Using the estimation results in Equation (6), we can now obtain the average partial effect (APE) of different human resources and R&D expenses on revenue.

Finally, using the parameter estimation results from Equations (6) or (10), we can estimate the life cycle curve function of the MCI as follows:

(9) $$\hat{ln\;REVENUE}={\hat{\delta }}_{0}+{\hat{\delta }}_{1}AGE+{\hat{\delta }}_{2}AGE^{2}$$

In this Equation, the value of ${\hat{\delta }}_{0}$ is estimated as follows in accordance with the translog model:

(10) $${\hat{\delta }}_{0}={\hat{\beta }}_{0}+{\hat{\alpha }}_{1}\bar{lnHUCAP}+{\hat{\beta }}_{1}\bar{lnDIRECT}+{\hat{\beta }}_{2}\bar{lnMANAGER}+{\hat{\gamma }}_{1}\bar{lnRD_{-1}}\;+{\hat{\alpha }}_{11}\bar{{\left(lnHUCAP\right)}^{2}}+{\hat{\beta }}_{11}\bar{{\left(lnDIRECT\right)}^{2}}+{\hat{\beta }}_{22}\bar{{\left(lnMANAGER\right)}^{2}}+{\hat{\gamma }}_{11}\bar{{\left(lnRD_{-1}\right)}^{2}}\;+{\hat{\beta }}_{12}\bar{lnDIRECT}\bar{lnMANAGER}+{\hat{\varepsilon }}_{11}\bar{lnHUCAP}\bar{lnRD_{-1}}\;+{\hat{\theta }}_{11}\bar{lnHUCAP}\bar{lnDIRECT}+{\hat{\theta }}_{12}\bar{lnHUCAP}\bar{lnMANAGER}\;+{\hat{\eta }}_{11}\bar{lnDIRECT}\bar{lnRD_{-1}}+{\hat{\eta }}_{21}\bar{lnMANAGER}\bar{lnRD_{-1}}$$

In this equation, $\bar{REGRESSOR}$ represents the mean value of the regressor. The value of ${\hat{\delta }}_{0}$ is estimated as follows in accordance with the Cobb–Douglas model:

(11) $${\hat{\delta }}_{0}={\hat{\beta }}_{0}+{\hat{\alpha }}_{1}\bar{lnHUCAP}+{\hat{\beta }}_{1}\bar{lnDIRECT}+{\hat{\beta }}_{2}\bar{lnMANAGER}+{\hat{\gamma }}_{1}\bar{lnRD_{-1}}$$

In Equation (9), the partial derivative of $\hat{ln\;REVENUE}\;\mathrm{with}\;\mathrm{respect}\;\mathrm{to}\;AGE$ is as follows:

(12) $$\partial \;\hat{ln\;REVENUE}\;/\partial \;AGE={\hat{\delta }}_{1}+2{\hat{\delta }}_{2}\;AGE$$

The above equation expresses the instantaneous change of the growth rate of the total revenue (*REVENUES*) of an MCE with respect to its years of establishment (*AGE*) and is essentially observing the evolution of the industry’s life cycle.

## 6. Empirical Results

### 6.1. Estimation Results of the Translog Revenue Function

The parameter estimation results of the translog revenue functions for SMMCEs are listed in Table 5. First, we used the null hypothesis established in Equation (7) to test for the existence of any interaction effect or effects from the second derivatives of the function between the independent variables. The F statistic values is 26.52 for SMMCEs, significantly rejecting the null hypothesis. This finding indicates that the translog model is more suitable than the Cobb–Douglas model for analyzing the revenue function of MCEs. This finding also implies that an interaction effect exists between production line personnel, technical personnel, managers, employee educational background, and R&D expenses at SMMCEs, which in turn affects the total revenue of MCEs. The team’s human capital emphasizes the heterogeneity [71,83,84] and diversity [85,86,87,88] of employees with different educational backgrounds and professional skills working together. If their different perspectives can help the team measure and manage risk, improve the team’s business vitality, and engender greater team creativity, it will have a positive impact on the enterprise’s total revenue [89,90,91]. In addition, employees with different educational backgrounds show different R&D efficiencies, which have different impacts on the cost-effectiveness of R&D expenses, and thus they affect the total revenue of MCEs. The above jointly verified Hypothesis 1 (H1) of this research.

Employing the parameter estimation results of the translog revenue function in Table 5 and Equations (6), we obtained the estimated the APE values of different human resources and R&D expenses on revenue, which are summarized in Table 6. It can be seen from Table 6 that the estimated APE values of human capital (*APE_HUCAP*) are significantly negative, which indicates that increasing the inputs on education (the proxy of human capital) decreases the actual revenue of a medical consumable enterprise. Hypothesis H2a cannot be verified. Further analysis revealed that the funds of SMMCEs were relatively limited. If there were large-scale investment in education, it would be bound to affect the capital investment of enterprises in other fields. When the revenue brought by education investment is less than the loss caused by insufficient investment in other fields, the enterprise will suffer losses. From the empirical results, Taiwan’s SMMCEs’ investment in education cannot maximize enterprise revenues. It can be seen from Table 6 that the estimated APE values of R&D expenses (*APE_R&D*) is significantly positive, which indicates that increasing R&D expenditure increased the actual revenue for a medical consumable enterprise. This empirical finding verified hypothesis H2c of this study.

With regard to the APE values of human resource quantity on revenue, the APE values of direct personnel (*APE_DIRECT*) and managers (*APE_MANAGER*) were all significantly positive. This finding indicates that manpower inputs have a driving effect on the performance of SMMCEs. This empirical finding verified hypothesis H2b of this study and was in accordance with the monotonic increase in manpower inputs in the revenue function.

### 6.2. Estimation Function and Estimation Curve of the Life Cycle of SMMCI

First, we use the parameter estimation results from Equations (9) and (10) to obtain the life cycle functions for SMMCEs, which are shown as Equation (13) below.

(13) $$\hat{ln\;REVENUE}=12.61412+0.05904AGE-0.00146AGE^{2}$$

For Equation (13), the partial derivatives of $\hat{ln\;REVENUE}\;\mathrm{with}\;\mathrm{respect}\;\mathrm{to}\;AGE$ are

(14) $$\partial \;\hat{ln\;REVENUE}\;/\partial \;AGE=0.05904-0.00292\;AGE$$

**Figure 1:**
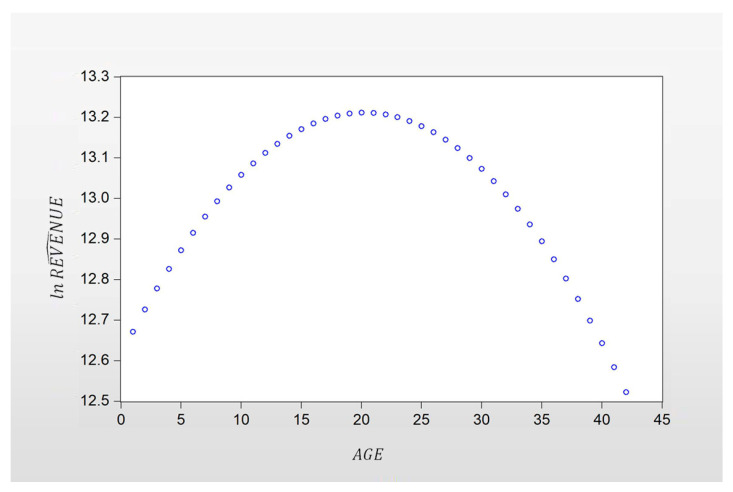
Life cycle curve of SMMCI.

Finally, Equation (13) was used to plot Figure 1, which showed the life cycle curves of the SMMCI. 

Equation (14) shows the growth rates of total revenue (*REVENUES*) corresponding to the years of establishment (*AGE*) of SMMCEs. It can be seen from Table 5, that the ${\hat{\delta }}_{2}$ value is 0.00292 and significant. Although these coefficients are seemingly very small, Wooldridge [92] believes that they are significant because they allow us to calculate the partial effect and to observe the variation in the total revenue growth rate with the years of establishment of MCEs. 

For SMMCEs, the estimation result of ${\hat{\delta }}_{1}$ was positive, and the estimation result of ${\hat{\delta }}_{2}$ was negative, indicating that the life cycle curve of MCEs was concave downward and had an inflection point. The concave downward life cycle curves are shown in Figure 1, and this result verified hypothesis 3 (H3) of this study. During the life cycle of a MCE, revenue grew quickly during the startup and growth stages, slowed during the maturity stage, and fell during the decline stage. The life cycle curves of the MCI we found are similar to the results of previous scholars’ research on corporate lifecycles [8].

## 7. Conclusions

With the increasing global demand for medical consumables, the MCI has made great progress in recent years, but it is also faced with market uncertainty. Whether from a long-term or short-term perspective, research on the development of the MCI has important practical significance. This research uses the translog revenue function to estimate the life cycle curve of Taiwan’s SMMCI, and explores the correlation between the total revenue of the MCI and R&D expenses and human resources inputs. The empirical results showed that there was an interactive influence between different human resources in the SMMCI as well as between human resources and R&D expenditure, which further affected the total revenue of the industry. At the same time, the total revenue increased with the increase of R&D and manpower inputs, and decreased with the increase of education investment. This means that for SMMCEs, the revenue brought by the limited resources invested in education could not cover the losses caused by other insufficient investments. Given that the sample period of this study was from 2009 to 2018, the corresponding empirical results may only be applicable to the short-term and medium-term. Therefore, we suggest that managers of SMMCEs consider appropriately reducing their investment in education in the short-to-medium term while simultaneously increasing their investment in the number of employees and R&D. The competent department of the MCI can refer to the results of this study to guide the SMMCEs to make rational investments in order to maximize their cost-benefit ratio.

This research was the first to use the revenue function in economic theory and empirical data of the MCI to study the life cycle curve function of the MCI. The curve of the industry life cycle showed that the SMMCI has different revenue growth in different life cycle stages. According to the trend of the curve, the revenue growth rate of the SMMCI is relatively high in the startup and growth stages. We suggest that enterprises should not focus on the growth of revenue at these stages but pay more attention to the assessment of the risk situation faced by enterprises as a means to improve their ability to resist risks. From Table 4, it can be seen that the correlation coefficient between the SMMCI’s investment in education and revenue is significantly negative, and from Table 6, it can be seen that the APE of education is significantly negative. All the above data showed that SMMCI’s investment in education will reduce revenue, and the reduction of revenue may result in enterprises facing the problem of capital chains, especially for the SMMCI at the start-up and growth stages, which has weak risk resistance to potential liquidity dilemmas. Therefore, strengthening the risk resistance ability and strengthening the risk awareness and risk management training of the enterprise management should be the industry’s strategic focus at this time, which is consistent with the results of research by Kaszuba-Perz and Czyzewska [93]. In the maturity stage of the SMMCI, although the industry’s total revenue was higher, the profitability begins to slow, and the industry gradually entered the decline stage. We suggest that the SMMCEs should change their emphasis from the enhancement of enterprise risk resistance to the growth of corporate profitability to slow the pace of the industry’s entering the decline stage. At the same time, industrial upgrading can be achieved through industrial transformation and development of high value-added businesses, thus extending the life cycle.

Our empirical results also helped to explain some phenomena observed in the MCI, such as differences in the total revenue of MCEs with different human resource structures, and the fact that the total revenue growth of MCEs followed the “rapid growth—slowing down—decline” stages. MCEs should examine the current stage of their life cycle with a forward-looking attitude, assess their fundamentals based on the characteristics of the stages of their life cycle, and establish appropriate development strategies to better measure and manage risk. In addirtion, they should selectively implement resource inputs and enterprise management according to specific life cycle stages to reduce the impact of the current uncertainty in the global medical consumables market on business operations. 

In addition to the human factors ($x_{1},x_{2}$) that are common to all industries, the important influencing factors considered in this study were employee education and R&D inputs according to the nature of MCI that requires a large amount of research and development inputs. As for other possible influencing factors, such as the parameters of the underlying production technology and possible fixed factors, it was difficult for us to take these factors into consideration due to the availability of data. This is also one of the limitations of this study. We call on scholars to conduct more comprehensive research if they can obtain relevant data, which will become one of the future research directions.

## Figures and Tables

**Table 1:** Summary of the numbers of samples in the selection.

Total companies on Taiwan Economic Journal from 2009 to 2018	300
Deletions:	
(A) Zero whole-year revenue	(29)
(B) Zero education background of employees	(10)
(C) Zero experience of employees	(4)
(D) Zero employees	(32)
(E) Zero research and development spending	(27)
Total companies of SMMCE data from 2009 to 2018	198

**Table 2:** Variable definitions.

Variable		Definition
Theoretical Variable	Proxy Variable	
*r*	*REVENUE*	Total revenue of medical consumables enterprises, expressed in million NT$
*h*	*HUCAP*	*HUCAP* represents the human capital acquired through specialized education. *EDUC* as a proxy variable of *HUCAP*
	*EDUC*	Employees’ average number of years of education
*x*_1_	*DIRECT*	Total number of production line employees and technical personnel
*x*_2_	*MANAGER*	Total number of management personnel
	*EMPLOYEE*	Total number of employees
*d*	*RD*	Total research and development spending
	*AGE*	Medical consumables enterprise’s years of establishment

**Table 3:** Descriptive statistics.

**Panel A:**	**2009 (*n* = 14)**						**2010 (*n* = 16)**					
**Variables**	**Mean**	**Median**	**Max**	**Min**	**Std. Dev.**	**Kurtosis**	**Mean**	**Median**	**Max**	**Min**	**Std. Dev.**	**Kurtosis**
*REVENUE*	NT$756.6	NT$588.7	NT$2392.4	NT$7.4	NT$719.1	3.6	NT$970.8	NT$689.3	NT$3099.3	NT$37.2	NT$826.9	3.8
*EDUC*	14.8	15.2	17.1	12.2	1.6	2.0	14.8	14.8	17.2	12.3	1.5	2.2
*DIRECT*	177.6	34.5	1155.0	3.0	315.3	7.8	210.7	77.0	1630.0	3.0	401.1	11.2
*MANAGER*	81.1	62.5	347.0	1.0	85.4	8.0	92.1	63.5	336.0	10.0	89.5	5.0
*EMPLOYEE*	258.7	96.5	1257.0	24.0	346.6	6.0	302.8	115.5	1733.0	38.0	423.7	9.3
*RD*	NT$37.3	NT$32.8	NT$173.8	NT$0.6	NT$42.1	9.2	NT$46.3	NT$31.6	NT$180.5	NT$2.1	NT$45.4	6.1
*AGE*	17.1	16.5	33.0	9.0	6.3	3.9	17.3	16.5	34.0	7.0	6.8	3.4
**Panel B:**	**2011 (*n* = 18)**						**2012 (*n* = 20)**					
**Variables**	**Mean**	**Median**	**Max**	**Min**	**Std. Dev.**	**Kurtosis**	**Mean**	**Median**	**Max**	**Min**	**Std. Dev.**	**Kurtosis**
*REVENUE*	NT$878.3	NT$548.7	NT$3458.6	NT$37.2	NT$880.0	5.2	NT$1110.6	NT$604.5	NT$4168.7	NT$0.3	NT$1203.7	4.0
*EDUC*	15.0	15.0	17.2	12.5	1.5	2.4	15.1	15.1	17.4	12.5	1.5	2.0
*DIRECT*	214.1	59.5	1932.0	2.0	450.8	13.2	247.1	59.5	2120.0	1.0	488.3	12.1
*MANAGER*	88.7	65.5	296.0	12.0	76.9	4.6	119.7	69.5	764.0	4.0	168.3	12.0
*EMPLOYEE*	302.7	111.5	2040.0	38.0	470.6	11.6	366.8	117.5	2231.0	10.0	567.9	7.7
*RD*	NT$44.4	NT$30.8	NT$164.3	NT$1.7	NT$42.4	4.9	NT$45.7	NT$33.4	NT$165.8	NT$0.8	NT$42.6	4.8
*AGE*	17.3	16.0	35.0	8.0	7.0	3.2	17.0	16.0	36.0	5.0	7.7	3.0
**Panel C:**	**2013 (*n* = 20)**						**2014 (*n* = 20)**					
**Variables**	**Mean**	**Median**	**Max**	**Min**	**Std. Dev.**	**Kurtosis**	**Mean**	**Median**	**Max**	**Min**	**Std. Dev.**	**Kurtosis**
*REVENUE*	NT$1179.1	NT$624.4	NT$5222.2	NT$61.3	NT$1436.0	6.0	NT$1270.9	NT$682.4	NT$5782.9	NT$26.5	NT$1600.1	6.1
*EDUC*	14.6	14.6	17.2	10.9	1.7	2.4	14.7	14.7	17.3	11.6	1.6	2.2
*DIRECT*	338.6	80.0	2946.0	1.0	689.2	11.7	324.3	83.5	2712.0	1.0	648.2	10.7
*MANAGER*	153.4	75.0	872.0	14.0	200.3	9.4	163.4	78.0	959.0	13.0	215.9	10.4
*EMPLOYEE*	492.0	130.5	3057.0	33.0	783.5	7.8	487.7	146.0	2825.0	30.0	759.8	7.2
*RD*	NT$55.9	NT$44.0	NT$159.4	NT$10.8	NT$40.4	3.6	NT$61.9	NT$44.4	NT$178.5	NT$8.2	NT$47.9	3.4
*AGE*	16.8	16.0	37.0	1.0	8.5	3.1	17.8	17.0	38.0	2.0	8.5	3.1
**Panel D:**	**2015 (*n* = 20)**						**2016 (*n* = 20)**					
**Variables**	**Mean**	**Median**	**Max**	**Min**	**Std. Dev.**	**Kurtosis**	**Mean**	**Median**	**Max**	**Min**	**Std. Dev.**	**Kurtosis**
*REVENUE*	NT$1320.2	NT$684.6	NT$6098.9	NT$0.1	NT$1679.6	6.3	NT$1282.0	NT$542.2	NT$6539.5	NT$1.6	NT$1809.6	6.7
*EDUC*	14.8	14.9	17.9	12.2	1.6	2.2	15.1	15.1	18.4	12.0	1.7	2.3
*DIRECT*	326.7	77.0	2919.0	6.0	683.6	11.3	332.2	68.0	3127.0	8.0	736.2	11.0
*MANAGER*	168.6	78.0	1041.0	9.0	234.2	10.3	143.8	73.0	800.0	4.0	183.3	8.2
*EMPLOYEE*	495.3	129.0	3076.0	22.0	809.9	7.6	476.0	125.0	3326.0	18.0	847.3	8.6
*RD*	NT$67.6	NT$40.4	NT$203.6	NT$5.7	NT$58.6	3.2	NT$69.2	NT$40.0	NT$221.3	NT$5.2	NT$59.0	3.3
*AGE*	17.6	17.0	39.0	3.0	9.1	2.7	17.4	17.0	40.0	4.0	9.5	2.6
**Panel E:**	**2017 (*n* = 20)**						**2018 (*n* = 20)**					
**Variables**	**Mean**	**Median**	**Max**	**Min**	**Std. Dev.**	**Kurtosis**	**Mean**	**Median**	**Max**	**Min**	**Std. Dev.**	**Kurtosis**
*REVENUE*	NT$1293.9	NT$534.5	NT$6417.9	NT$1.9	NT$1735.0	6.2	NT$1504.3	NT$629.0	NT$7389.2	NT$1.0	NT$1995.0	6.0
*EDUC*	15.1	15.2	18.2	12.0	1.6	2.5	15.2	15.3	18.0	12.5	1.6	2.1
*DIRECT*	334.7	86.0	3389.0	6.0	762.3	12.9	369.4	69.0	3651.0	4.0	827.3	12.5
*MANAGER*	170.7	69.0	1111.0	6.0	242.9	10.9	183.0	66.0	1241.0	9.0	274.3	10.8
*EMPLOYEE*	505.3	123.0	3597.0	23.0	904.5	8.8	552.5	148.0	3904.0	29.0	997.3	8.5
*RD*	NT$70.0	NT$42.2	NT$216.3	NT$5.1	NT$66.0	3.3	NT$70.9	NT$41.9	NT$222.6	NT$5.3	NT$66.3	3.2
*AGE*	18.4	18.0	41.0	5.0	9.5	2.6	19.4	19.0	42.0	6.0	9.5	2.6
**Panel F:**	**2019–2018 (*n* = 20)**											
**Variables**	**Mean**	**Median**	**Max**	**Min**	**Std. Dev.**	**Kurtosis**						
*REVENUE*	NT$1185.4	NT$618.1	NT$7389.2	NT$0.1	NT$1488.0	8.2						
*EDUC*	14.9	15.0	18.4	10.9	1.6	2.3						
*DIRECT*	296.0	76.0	3651.0	1.0	631.4	14.8						
*MANAGER*	141.0	70.5	1241.0	1.0	195.5	14.6						
*EMPLOYEE*	437.0	124.0	3904.0	10.0	738.2	10.9						
*RD*	NT$58.5	NT$39.9	NT$222.6	NT$0.6	NT$53.2	4.4						
*AGE*	17.7	17.0	42.0	1.0	8.3	3.0						

**Table 4:** Correlation matrix for dependent and independent variables (*p*–values in parentheses).

ty	REVENUE	EDUC	DIRECT	MANAGER	EMPLOYEE	$RD_{-1}$	AGE
**REVENUE**	1.0000						
	-----						
**EDUC**	−0.4670	1.0000					
	(0.0000)	-----					
**DIRECT**	0.8920	−0.4825	1.0000				
	(0.0000)	(0.0000)	-----				
**MANAGER**	0.7273	−0.3113	0.4377	1.0000			
	(0.0000)	(0.0000)	(0.0000)	-----			
**EMPLOYEE**	0.9556	−0.4951	0.9712	0.6391	1.0000		
	(0.0000)	(0.0000)	(0.0000)	(0.0000)	-----		
$RD_{-1}$	−0.0198	0.0916	−0.0721	−0.0394	−0.0721	1.0000	
	(0.7822)	(0.2004)	(0.3138)	(0.5830)	(0.3138)	-----	
**AGE**	0.2481	−0.4101	0.3184	−0.0621	0.2559	0.1895	1.0000
	(0.0004)	(0.0000)	(0.0000)	(0.3846)	(0.0003)	(0.0077)	-----

All variables are as defined in Table 2.

**Table 5:** Estimation of translog revenue function for data pooled over 2009–2018 (t–statistics in parentheses).

$\;lnREVENUE=\beta_{0}+\alpha_{1}lnHUCAP+\beta_{1}lnDIRECT+\beta_{2}lnMANAGER+\gamma_{1}lnRD_{-1}$ $+\alpha_{11}{\left(lnHUCAP\right)}^{2}+\beta_{11}{\left(lnDIRECT\right)}^{2}+\beta_{22}{\left(lnMANAGER\right)}^{2}+\gamma_{11}{\left(lnRD_{-1}\right)}^{2}$ $+\beta_{12}\left(lnDIRECT\right)(lnMANAGER)+\varepsilon_{11}(lnHUCAP)(lnRD_{-1})$ $+\theta_{11}(lnHUCAP)(lnDIRECT)+\theta_{12}(lnHUCAP)(lnMANAGER)$ $+\eta_{11}(lnDIRECT)(lnRD_{-1})+\eta_{21}(lnMANAGER)(lnRD_{-1})$ $+\delta_{1}AGE+\delta_{2}AGE^{2}$			(6)
**Variables**	**Coefficient**	**Variables**	**Coefficient**
	***t*** **-Statistic**		***t*** **-Statistic**
Intercept	180.80080	(ln*DIRECT*)(ln*MANAGER*)	−0.54768 ***
	(2.79722)		(−6.35094)
ln*HUCAP*	−131.4221 ***	(ln*HUCAP*)(ln$RD_{-1}$)	−0.13573 ***
	(−2.89204)		(−3.73192)
ln*DIRECT*	−20.25925 ***	(ln*HUCAP*)(ln*DIRECT*)	0.16602 ***
	(−6.71300)		(2.61818)
ln*MANAGER*	3.60965 **	(ln*HUCAP*)(ln*MANAGER*)	7.65354 ***
	(1.73059)		(7.68211)
ln$RD_{-1}$	9.39778 ***	(ln*DIRECT*)(ln$RD_{-1}$)	−1.12340
	(3.50342)		(−1.57789)
(ln*HUCAP*) ^2^	25.17747 ***	(ln*MANAGER*)(ln$RD_{-1}$)	−3.62483 ***
	(3.02896)		(−4.30462)
(ln*DIRECT*) ^2^	0.38241 ***	*AGE*	0.05903 **
	(9.43003)		(2.44598)
(ln*MANAGER*) ^2^	0.10776	*AGE* ^2^	−0.00145 **
	(1.65134)		(−2.39902)
(ln$RD_{-1}$) ^2^	0.02610		
	(0.92229)		
Adjusted R–squared	0.868		
System degrees of freedom	198		
*Test of log–linear specification* ($H_{0}:{\;\alpha }_{11}=\beta_{\mathrm{ij}}=\gamma_{11}=\theta_{1i}=\varepsilon_{11}=\eta_{i1}=0;\;i,\;j=1,\;2$)			
*F*–statistic	26.52		
Significance level	0.000		

***, **, * Denotes significantly difference from zero at the 1%, 5%, and 10% levels, respectively (two–tailed test). The model was estimated using ordinary least squares. All variables are as defined in Table 2.

**Table 6:** APE of human resources and R&D expenses on revenue.

APE	APE Estimated Value	Significance Test
		$H_{0}:{\hat{\beta }}_{2}={\hat{\beta }}_{6}={\hat{\beta }}_{13}={\hat{\beta }}_{14}={\hat{\beta }}_{15}=0$
*APE_HUCAP*	−2.80	*F*–statistic = 15.43
		Significance level = 0.00
		$H_{0}:{\hat{\beta }}_{3}={\hat{\beta }}_{7}={\hat{\beta }}_{10}={\hat{\beta }}_{11}={\hat{\beta }}_{13}=0$
*APE_DIRECT*	0.33	*F*–statistic = 37.62
		Significance level = 0.00
		$H_{0}:{\hat{\beta }}_{4}={\hat{\beta }}_{8}={\hat{\beta }}_{10}={\hat{\beta }}_{12}={\hat{\beta }}_{14}=0$
*APE_MANAGER*	0.86	*F*–statistic = 36.26
		Significance level = 0.00
		$H_{0}:{\hat{\beta }}_{5}={\hat{\beta }}_{9}={\hat{\beta }}_{11}={\hat{\beta }}_{12}={\hat{\beta }}_{15}=0$
*APE_* $RD_{-1}$	0.08	*F*–statistic = 7.69
		Significance level = 0.00

## Data Availability

All relevant data are within the manuscript.
